# Spinal infection with intraspinal abscess or empyema and acute myelopathy: comparative analysis of diagnostics, therapy, complications and outcome in primary care

**DOI:** 10.1007/s00068-022-02001-1

**Published:** 2022-06-03

**Authors:** Martin Kreutzträger, Tom Lübstorf, Axel Ekkernkamp, Christian Blex, Jan M. Schwab, Marcel A. Kopp, Thomas Auhuber, Grit Wüstner, Thomas Liebscher

**Affiliations:** 1 Treatment Centre for Spinal Cord Injuries, BG Hospital Unfallkrankenhaus Berlin, Trauma Hospital Berlin, Warener Straße 7, 12683 Berlin, Germany; 2 Department of Neurology and Experimental Neurology, Spinal Cord Injury Research, Charité – Universitätsmedizin Berlin, Berlin, Germany; 3 Trauma Surgery and Orthopedics Clinic, BG Hospital Unfallkrankenhaus, Berlin, Germany; 4 Spinal Cord Injury Division, Department of Neurology, Belford Center for Spinal Cord Injury, The Ohio State University, Wexner Medical Center, Columbus, OH 43210 USA; 5 Department of Neuroscience, The Neurological Institute, The Ohio State University, Wexner Medical Center, Columbus, OH 43210 USA; 6 Department of Physical Medicine and Rehabilitation, The Neurological Institute, The Ohio State University, Wexner Medical Center, Columbus, OH 43210 USA; 7 Berlin Institute of Health, QUEST – Center for Transforming Biomedical Research, Berlin, Germany; 8 Medical Management, Trauma Hospital Berlin, Berlin, Germany; 9 University of the German Statutory Accident Insurance (HGU), Bad Hersfeld, Germany; 10 BG Hospital Unfallkrankenhaus Berlin, Berlin, Germany

**Keywords:** Spondylodiscitis, Spinal surgery, Intra-spinal empyema, Intra-spinal abscess, Acute spinal cord injury, Treatment costs

## Abstract

**Introduction:**

This study on pyogenic spinal infections with intraspinal epidural involvement (PSI +) compared the outcome of patients with spinal cord injury (SCI) to those without (noSCI) taking diagnostic algorithm, therapy, and complications into account.

**Methods:**

Patients were enrolled in an ambispective study (2012–2017). Diagnostic and therapeutic algorithms, complications, and neurological outcome were analyzed descriptively. Survival was analyzed applying Kaplan–Meier method and Cox regression.

**Results:**

In total, 134 patients with a median (IQR) age of 72 (61–79) years were analyzed. Baseline characteristics were similar between the SCI (*n* = 55) and noSCI (*n* = 79). A higher percentage of endocarditis (9% vs. 0%; *p* = 0.03) was detected in the noSCI group. The majority (81%) received combinatorial therapy including spinal surgery and antibiotic treatment. The surgery complication rate was 16%. At discharge, improvement in neurologic function was present in 27% of the SCI patients. Length of stay, duration of ventilation and the burden of disease-associated complications were significantly higher in the SCI group (e.g., urinary tract infection, pressure ulcers). Lethality risk factors were age (HR 1.09, 95% CI 1.02–1.16, *p* = 0.014), and empyema/abscess extension (≥ 3 infected spinal segments, HR 4.72, 95% CI 1.57–14.20, *p* = 0.006), dominating over additional effects of Charlson comorbidity index, SCI, and type of treatment. The overall lethality rate was 11%.

**Conclusion:**

PSI + are associated with higher in-hospital mortality, particularly when multiple spinal segments are involved. However, survival is similar with (SCI) or without myelopathy (noSCI). If SCI develops, the rate of disease complications is higher and early specialized SCI care might be substantial to reduce complication rates.

## Introduction

Pyogenic spinal infections with intraspinal epidural involvement (PSI +) represent a subgroup whose diagnosis, treatment, and outcome have been rarely studied [1]. The rising incidences of spinal infections in recent decades are due, among other factors, to an aging population, an increase in patients’ secondary illnesses, and the increasing use of immunosuppressants [2]. High mortality rates of pyogenic spinal infections mounting up to 20% have been reported [3, 4].

In case of severe infection, intraspinal abscesses (ISA) or empyema (ISE) may occur [5]. The involvement of the spinal canal leads to acute spinal cord injury (SCI) in up to 50% of cases [6].

There are only a few evidence-based medicine level studies published for conservative therapies, otherwise most recommendations are based on studies with a low level of evidence [1]. Treatment algorithms of spinal infections are controversial because of multimodal and individualized therapy resulting in inhomogeneous treatments and outcome data [7]. In case of SCI and sepsis, surgery is recommended [8] as well as for ISA or ISE due to the risk of SCI [6–8].

The aim of this work is to compare diagnostics, treatment algorithm, and clinical and socioeconomic outcome of patients with pyogenic spinal infections with intraspinal epidural involvement (PSI +) with or without SCI.

## Methods

### Patients

All patients with PSI + were consecutively enrolled in a monocentric study with an ambispective design from January 2012 to September 2017. The study collected prospective data on the clinical management and outcome of patients with PSI data from May 2015 until September 2017. The prospective dataset was completed by collecting dataset of the same structure retrospectively from January 2012 to May 2015. Intra-spinal affection was defined as the presence of an ISA or ISE.

On admission and end of acute care, patients were classified as having no SCI (noSCI) or SCI (SCI) according to the International Standards for Neurological Classification of SCI (ISNCSCI). Baseline characteristics, i.e., sex, age, body mass index (BMI), and the Charlson comorbidity index (CCI), were recorded on admission [9].

The length of stay in the inpatient unit, the intensive care unit (ICU), and ventilation times were assessed. Treatment cost data stored in the cost and activity accounting system "WICO" (Cerner Health Services) were calculated using the software "eisTIKAKUT" (KMS AG, Unterhaching, Germany) as provided by the Institute for the Hospital Remuneration System (InEK, Siegburg, Germany). All data stored in the hospital information systems “Medico Portal” (Cerner Health Services, Idstein, Germany), “ICM Portal” (Drägerwerk, Lübeck, Germany) or the radiograph system “IntelliSpace PACS Enterprise” (Philips Healthcare Informatics, Hamburg, Germany) were compiled for statistical evaluation using a versioned database.

The study was approved by the Ethics Committee of Charité—Universitätsmedizin Berlin, Germany, EA2/015/15. The study was carried out in compliance with Declaration of Helsinki.

### Diagnostics

Laboratory parameters were assessed on admission and at the end of acute care. Blood cultures were performed on admission before the start of antibiotic treatment. A minimum of three microbiological specimens were obtained during each surgical procedure.

The entire spine was examined using contrast enhanced magnetic resonance imaging. The extended diagnostics also included contrast enhanced computed tomography of the thorax and abdomen. To exclude a dental focus, a pantomogram was performed and a specialist oral and maxillofacial surgeon was consulted. If a potential focus of infection was found, dental and maxillofacial surgery was performed.

The search for a cardiac focus in terms of endocarditis was performed by echocardiography. Specialist evaluation was performed by cardiologists and, in the case of a finding worthy of surgery, by cardiac surgeons.

### Antibiotic therapy

Antibiotic treatment was always determined by consultation of microbiologists. Antibiotic therapy was started after intraoperative swabs were obtained, except in cases of sepsis according to CDC criteria [10]. In all cases without known evidence of pathogens, empirical therapy with cefuroxime and fosfomycin was given intravenously for two weeks. Subsequently, patients received sulfamethoxazole/trimethoprim and rifampicin orally. With evidence of a pathogen, therapy was switched to match resistance. With purely conservative therapy, antibiotic therapy was given for at least six weeks. Antibiotics were administered for at least three months after surgical stabilization.

### Surgical therapy

In all patients, the indication for surgery was assessed via clinical, radiological and neurological criteria. The intentions of surgery were infection control, prevention of further spreading and recurrence, and avoidance or improvement of neurologic deficits. For this reason, laminectomy or, in the cervical spine, ventral opening of the spinal canal was always performed. In cases of extensive ISE, laminectomy was performed at the point of greatest spinal narrowing followed by spinal canal irrigation with saline through a 4.5 Charrière catheter. Patients who refused to give consent for surgical intervention or were multimorbid with high risks not to survive the procedure, were considered for conservative therapy.

Stabilization was performed if there was osseous involvement or laminectomy over more than one spinal segment. Dorsoventral stabilization of the spine was performed if one of the following criteria was met: segmental spinal kyphosis > 15°, vertebral body collapse > 50%, or segmental translation > 5 mm [11]. Surgery was not performed in patients who were unfit for surgery or unwilling to undergo surgery.

### Complications and outcome

Disease-associated complications, such as pneumonia, thromboembolism, or pressure ulcers, can occur in PSI + without and with SCI during treatment. Complications of spinal surgery were defined as any unexpected adverse event related to the spinal surgery [12]. The clinical outcome parameter analyzed for the SCI group was neurological improvement in the ASIA impairment scale (AIS) between hospital admission and the end of acute treatment. Clinical outcome parameters analyzed for both groups were: (i) end-of-acute-care criteria (leukocytes within normal range, (ii) no fever, (iii) adequate low-pain mobility of at least 4 h per day, (iv) non-irritant wound conditions or (v) death during initial treatment.

### Statistical analysis

Continuous variables were reported as median and quartiles and compared using the Mann–Whitney *U* test. Categorical variables were reported as absolute and relative frequencies and compared using the *χ*^2^ test. The association of the number of affected spine segments with in-hospital mortality was analyzed using the Kaplan–Meier method. Patients were censored at discharge or at 90 days at the latest. The number of affected segments was dichotomized into 1–2 Segments vs. ≥ 3 segments. Groups were compared using the log-rank test. In addition, a Cox regression model was calculated under the proportional hazard assumption and adjusted for age, sex, CCI and SCI status. Patients who died during hospitalization were excluded from the analysis of length of stay and treatment costs. All tests were two-sided, and the statistical significance level was set to < 0.05. Explorative p values should be interpreted cautiously, as no adjustment for multiple testing was performed. Data processing and statistical analysis were done using the software SPSS (Version 27.0).

## Results

**Fig. 1:**
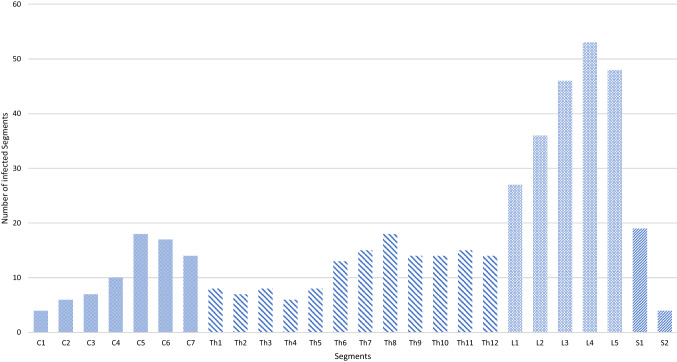
Infected spinal segments. The total number of involvements for each spinal segment is shown. The small dotted bars for the cervical, the striped bars for the thoracic and the squared bars for the lumbar/sacral segments. C, cervical; Th, thoracic; l, lumbar, s, sacral

In total, 134 patients were included in the study with a median (IQR) age of 72 (61–79). The noSCI group comprised 79 patients (59%) and the SCI group 55 (41%). Sex, BMI and CCI were similar between the groups (Table 1). PSI + was present in 106 patients (79%) with at least one affected spinal segment and an ISA or ISE. A solitary ISE was detected in 28 patients (21%). The number of infected spinal segments had three main localizations; the lower cervical, the middle thoracic, and the entire lumbar spine (Fig. 1). The hospital admission to surgery time interval was significantly increased in the noSCI group with a median (IQR) of 41 (21–141) hours compared to 28 (17–50) hours in the SCI group. Length of stay was significantly shorter in the noSCI group, as were the mechanical ventilation times. In contrast to the total length of stay, ICU treatment duration was similar for both groups (Table 2). The cost analysis of total expenses for acute care, revealed a significant difference between the noSCI group with a median (IQR) of 24,476 (18,525–47,741) Euros and the SCI group with 70,734 (35,794–92,482) Euros (*p* = 0.001).

**Table 1:** Baseline characteristic and Diagnostics

	Total *n* = 134	noSCI group *n* = 79 (59%)	SCI group *n* = 55 (41%)	*p* value
Baseline characteristics				
Age; median (IQR)	72 (61–79)	72 (60–78)	72 (62–80)	0.811
Gender (female: male); *n* (%)	64: 70 (47.8: 52.2)	38: 41 (48: 52)	26: 29 (47: 53)	
BMI; median (IQR)	26.6 (23.8–30.1)	27.4 (24.8–30.9)	26.1 (23.4–29.4)	0.122
CCI; median (IQR)	3 (2–6)	3 (1.8–6)	3 (2–6)	0.961
AIS at admission (A:B:C:D:E); *n* (%)	7:5:11:32:79 (5:4:8:24: 59)	0:0:0:0:79 (0:0:0:0:100)	7:5:11:32:0 (13:9:20:58)	–
Neurological level at admission (cervical:thoracic:lumbar); *n* (%)	–	–	11:10:34 (20:18:62)	–
Spinal infection with abscess; *n* (%)	106 (79.1)	64 (81)	42 (76.4)	0.525
Spinal infection with empyema; *n* (%)	28 (20.9)	15 (19)	13 (23.6)	0.525
Diagnostic results				
CRP in mg/l at admission in median (IQR)	143 (73.3–212.1)	152.7 (76.3–212.3)	104.8 (59.1–212.1)	0.554
CRP in mg/l at discharge in median (IQR)	23.8 (12.2–58.8)	29.2 (16.8–64.3)	15.1 (6.4–35.3)	0.110
Leukocytes in gpt/l at admission in median (IQR)	11.6 (8.3–15.0)	11.9 (8.4–14.7)	11.1 (7.4–15.5)	0.795
Leukocytes in gpt/l at discharge in median (IQR)	6.5 (4.9–8.2)	6.6 (5.0–8.7)	6.4 (4.9–8.2)	0.134
Positive blood cultures; *n* (%)	52 (38.8)	34 (43.0)	18 (32)	0.153
Proof of infection in Echocardiography; *n* (%)	7 (5.2)	7 (8.9)	0 (0)	0.030*
Proof of infection in dental examination; *n* (%)	14 (10.4)	10 (12.7)	4 (7.3)	0.749

*AIS* ASIA impairment scale, *BMI* body mass index, *CCI* charlson comorbidity index, *CRP* C-reactive protein, *IQR* interquartile range, *noSCI* no spinal cord injury, *SCI* spinal cord injury

**Table 2:** Timelines and treatment durations

	noSCI group *n* = 79 (%)	SCI group *n* = 55 (%)	*p* value
Red-flag until admission in days; median (IQR)	23 (11–40)	17 (9–31)	0.586
Time admission to surgery in hours; median (IQR)	41 (21–141)	28 (16.8–50)	0.042
Duration of treatment in intensive care unit in days; median (IQR)	3 (1–10.8)	4.5 (1.8–16.5)	0.316
Duration of mechanical ventilation in hours; median (IQR)	120 (96–120)	780 (564–1022.3)	0.034*
Admission to discharge in days; median (IQR)	23 (18–45.5)	56.5 (31.3–95.5)	0.001***

*IQR* interquartile range, *noSCI* no spinal cord injury, *SCI* spinal cord injury

### Diagnostics

Laboratory diagnostics of CRP and leukocyte count at admission and discharge were not different in both groups (Table 1). Overall, 90 patients (67%) had a pathogen detected in blood cultures (bacteremia) or intraoperative specimens (Table 3). Echocardiography revealed pathological findings only in the noSCI group (*n* = 7, 9%; *p* = 0.03) (Table 1).

**Table 3:** Distribution of pathogen spectrum

Pathogen	Frequency of detection	
	*n*	%
*Staphylococcus*	68	64.8
*Streptococcus*	11	10.8
Anaerobes	7	6.7
MRSA	7	6.7
*Enterococcus*	6	5.7
Enterobacteriaceae	2	1.9
MRGN	2	1.9
Pseudomonads	2	1.9

*MRSA* methicillin-resistant *Staphylococcus aureus*, *MRGN* multi-resistant gram-negative pathogens

### Therapy

*Conservative therapy* was applied in nine patients (16%) in the SCI group compared to 16 patients (20%) in the noSCI group. A comparative analysis revealed that the conservative treatment group and the surgical group were different in age [median (IQR) 74 (70–81.5) vs.71 (60–78); *p* = 0.023] and CCI [5 (2–7.5) vs.3 (1–6); *p* = 0.037] but were not different in sex and BMI. In patients receiving conservative therapy, antibiotic treatment lasted for a total of six weeks in eight (32%) cases, three months in 14 (56%) patients, and for longer than three months in three (12%) patients.

*Combinational therapy including spinal surgery in conjunction with local/spinal irrigation* was performed in 46 (84%) in the SCI group versus 63 patients (80%) in the noSCI group. The surgical approach was dorsal in 90 cases (83%), ventral in two cases, and combined dorsoventral in 17 cases (15%). Revision surgery was required in 17 cases (16%) due to mal-positioning (4%), persistent spine infection (5%), wound secretion (4%) or hematoma (4%). Antibiotic therapy was administered three months in 104 patients (95%), and longer than 3 months in 5 patients (5%).

### Complications and outcome

Disease-associated complications, such as urinary tract infection, pressure ulcers and circulatory dysregulation, were significantly higher in the SCI group (Table 4), whereas no difference was observed for thromboembolic events and pneumonia.

**Table 4:** Disease-associated complications

	noSCI group *n* = 79 (%)	SCI group *n* = 55 (%)	*p* value
Disease-associated complications			
Urinary tract infections	19 (24.1)	24 (43.6)	0.024*
Decubitus	3 (3.8)	9 (16.4)	0.027*
Pneumonia	11 (13.9)	11 (20)	0.356
Thromboembolism	1 (1.3)	0 (0)	1.000
Gastrointestinal inflammation	14 (17.7)	14 (25.5)	0.289

*noSCI* no spinal cord injury, *SCI* spinal cord injury

**Fig. 2:**
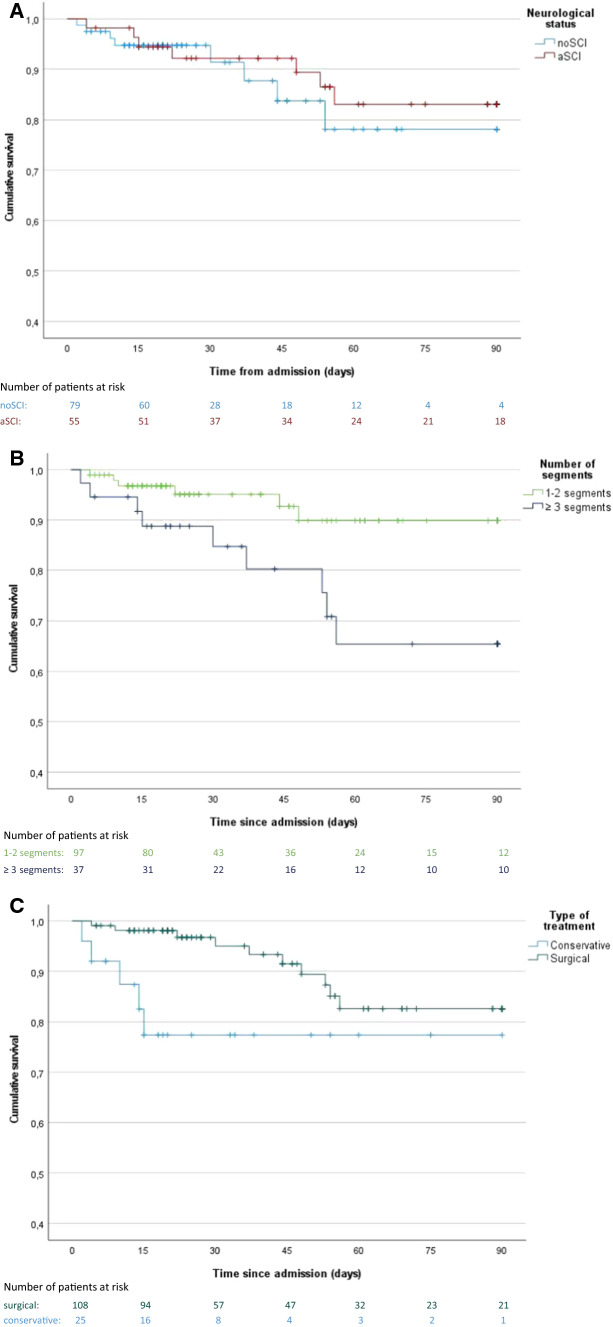
Survival analysis. Kaplan–Meier curves depicting the cumulating survival in days after admission comparing groups of **A** noSCI (without spinal cord injury) (light blue) and SCI (acute spinal cord injury) (red), **B** pyogenic spinal infection affecting 1–2 spinal segments (green) or ≥ 3 spinal segments (dark blue), and **C** Conservative treatment (cyan) or combined surgical and antibiotic treatment (dark green). Groups were compared using the log-rank test

Overall, a lethal outcome during of PSI + occurred in 15 patients (11.2%). The Kaplan–Meier analysis of cumulative survival during initial treatment, revealed no difference between noSCI and SCI groups (Fig. 2a), whereas a higher number of affected spinal segments was significantly associated with higher mortality (Fig. 2b), also the conservative treatment group had significantly higher cumulative mortality (Fig. 2c).

The bivariate (unadjusted) Cox regression model identified a number of ≥ 3 segments involved (HR (95% CI), 3.45 (1.22–9.72), *p* = 0.018), age per 1-year increase (HR (95% CI), 1.10 (1.03–1.18), CCI (HR (95% CI), 1.19 (1.01–1.40), *p* = 0.033) and the type of treatment (conservative) (HR (95% CI), 3.39 (1.12–10.06), *p* = 0.028) as risk factors for lethality. In the multivariable (covariate-adjusted) Cox-regression model, the number of ≥ 3 segments involved (HR (95% CI), 4.72 (1.57–14.20), *p* = 0.006), and age per 1-year increase were reaching statistical significance as risk factors for lethality (HR (95% CI), 1.09 (1.02–1.16), *p* = 0.014), whereas the effects of CCI and type of treatment were becoming smaller (Table 5).

**Table 5:** Survival analysis

	Bivariate model				Multivariate model			
	*n*	HR	95% CI	*p* value	*n*	HR	95% CI	*p* value
Number of infected spinal segments (≥ 3, ref 1–2)	134	3.45	1.22–9.72	0.018*	133	4.72	1.57–14.20	0.006**
Age (per 1-year increase)	134	1.10	1.03–1.18	0.003**		1.09	1.02–1.16	0.014*
CCI (per 1-point increase)	133	1.19	1.01–1.40	0.033*		1.11	0.91–1.36	0.305
Gender (male ref.. female)	134	0.93	0.34–2.56	0.882		0.84	0.27–2.58	0.755
Neurological deficits (SCI, ref. noSCI)	134	0.77	0.27–1.16	0.615		0.44	0.14–1.41	0.168
Type of treatment (conservative, ref. surgical)	133	3.39	1.12–10.06	0.028*		2.01	0.64–6.26	0.229

*CCI* charlson comorbidity index, *CI* confidence interval, *IQR* interquartile range, *HR* hazard ratio, *noSCI* no spinal cord injury, *ref* reference, *SCI* spinal cord injury

**Fig. 3:**
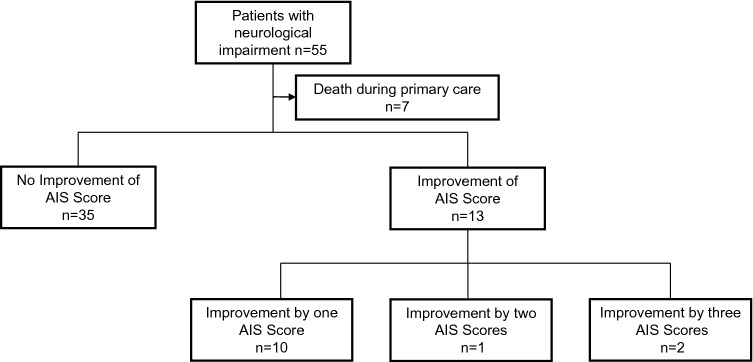
Neurological outcome. Chart for the neurological outcome of the SCI group according to the ASIA Impairment Scale (AIS). Death was observed in seven patients in this group during treatment, whereas 13 patients showed an improvement of at least von AIS Score

The SCI group comprised more motor incomplete SCI (AIS C: 20%, AIS D: 58%) than motor complete SCI (AIS A: 13%, AIS B: 9%) cases at admission. The neurological level was prevailingly lumbosacral (lumbosacral: 60%, thoracic: 20% and cervical: 20%). Improvement of the neurological status until discharge was observed in 13 of 48 patients (27%) (Fig. 3).

## Discussion

The baseline characteristics of PSI + with advanced age, polymorbidity, and immunosuppression in this study were comparable to the literature [13–15]. Our patients had a median CCI score of 3 indicating that both polymorbid and elderly people can be affected by PSI + .

Likewise, a typical pattern with the increase of CRP and leukocytes was shown without clear differences in both groups. In contrast, Lemaignen et al. demonstrated an increased risk for the occurrence of neurological deficits with a CRP value > 150 mg/l [4].

Echocardiography in this study demonstrated a statistically higher rate of cardiac infection events in the noSCI group. The proportion of cardiac infectious events is significantly lower (6%) compared with the literature (14–37%), probably due to a relatively short “red-flag” to admission time [1]. Echocardiography should always be performed in cases of spondylodiscitis with ISA or ISE, as well as an assessment of dental and jaw status [16].

Overall, a pathogen was detected in 67% in this study versus 81% of cases in other studies [17, 18]. For the appropriate therapy of a spinal infection without pathogen detection, there is no empirical evidence so far. Antibiotics should in any case cover the spectrum of staphylococci, since *Staphylococcus aureus* is detected in 30–80% of cases of spondylodiscitis [13, 19].

Antibiotic therapy periods are reported to range between 3 and 12 weeks, with duration of intravenous application of 4–6 weeks [7, 14, 20–22]. Bernard et al. demonstrated no inferiority of treating patients for 6 versus 12 weeks, with exceptions for age > 75 years, immunosuppression, neurological deficits, diabetes, and endocarditis [23]. Antibiotic therapy in our study was administered for six weeks in 32% and for three months or longer in 68% of the cases treated conservatively. Reasons for the longer treatment were mainly persistent infection parameters. In combination with surgery, three weeks of parenteral antibiotic therapy does not result in an increased number of reinfections compared with more than three weeks of therapy in patients without risk factors, such as infection with MRSA, diabetes, positive blood cultures, or ISA. [24] In our patients, due to the risk factors of ISA or ISE, age, comorbidities and the osteo-synthesis material, antibiotic administration was scheduled for at least three months in 99% of the cases undergoing spine surgery.

Surgical treatment is usually recommended in case of deformity, persistent or increasing infection, presence of SCI, and intraspinal process [18, 25–27]. It should be performed as soon as possible within 36–72 h after first evidence of neurological symptoms, as practiced in our study with a median admission to surgery interval of 28 h [28–30]. Early surgery is reported to lower the risk of treatment failure or neurologic deterioration in spondylodiscitis more effectively than antibiotic therapy alone [31]. Various surgical procedures are described like ventral, dorsal or combined dorsoventral procedures following the principles of infection clearance, abscess or empyema relief and, if necessary, stabilization, all of which are lacking evidence generated in prospective randomized controlled trials [8, 27]. Debridement of the ventral site of infection is considered necessary by many authors [19, 30, 32, 33].

In our study, surgery was done in 81% of all cases. Due to patients’ refusal for operation or Inability to operate due to general condition, 19% were treated conservatively. In 82% of the cases undergoing surgery, a dorsal approach with laminectomy and, if necessary, with stabilization was performed. The dorsal-only approach with fusion without ventral debridement did not result in reinfection in a study with 48 patients at a mean follow-up of 64 months [34]. In contrast, Lerner et al. concluded that surgical debridement via dorsoventral surgery is essential [26]. We do not consider the ventral debridement to be without alternatives in spondylodiscitis with ISA or ISE [11]. Here, the focus is on relief and pathogen reduction. In this study, the purely dorsal approach demonstrated infection control at the end of acute treatment.

Some disease-associated complications were significantly increased in the SCI group. The high proportion of urinary tract infections could be related to inadequate bladder management and targeted antibiotic therapy for spinal infections, which does not cover the pathogen spectrum of urinary tract infections. The higher rate of pressure ulcers in the SCI group was due to inadequate mobility. Notably, there was no significant difference between the regarding pneumonia and thromboembolism. In particular, the significantly longer ventilation time in the SCI group did not result in a higher rate of pneumonia, this finding is probably attributable to prophylaxis of SCI-associated pneumonia by early mobilization and physiotherapy.

In the SCI group, improvement in neurological function was demonstrated in 27% of the cases. Compared to the literature, persistent impairment with sensory deficits up to 90% and bladder dysfunction up to 50% have been described [17, 27, 35, 36].

The mortality rate in this study was 11.2% with no difference between the groups with and without SCI. The literature data on mortality of patients with ISA and ISE vary; mortality rates of 10–20% have been described [3, 37, 38]. In a bivariate, unadjusted Cox regression model, risk factors for mortality were the number of ≥ 3 affected spinal segments, older age, conservative treatment, and CCI. In the multivariate model, the number of affected spinal segments and age reached statistical significance. The effects of conservative treatment and CCI, however, were considerably smaller when the analysis was covariate adjusted. Therefore, it can be assumed that the reasons for a decision against surgical therapy, particularly older patient’s age and/or a poor underlying health condition were superimposing effects of the type of therapy (surgical vs. conservative). Thus, conclusions on therapy and/or CCI-related survival remain preliminary. Of note, this study was not designed to analyze treatment effects in the first place. However, the observed differences in survival between surgical vs. conservative treatment warrant further investigation in larger multicenter studies. Regarding health conditions, an association between a mean number of six comorbidities and mortality has been demonstrated by others in large patient populations with ISA +  [39]. In summary, the strongest risk factors for mortality during first hospitalization in our study were increasing age and ≥ 3 spinal segments affected.

Based on the subgroup PSI + described in this study and the high rate of patients with neurological deficits (44%), we consider the relatively low lethality rate of 11.2% is possibly indicating the effectiveness of our extended diagnostic algorithm and multimodal treatment concept.

Length of stay and ventilation time were clearly higher in the SCI group. According to the health economic calculations, this is associated with approximately 2.5-fold higher treatment costs in the SCI group. This health economic aspect should be considered in future health services research on improved strategies for the management of PSI + .

Limitations of this study relate to the ambispective and single-center design. However, as the patients were consecutively enrolled, the risk for selection bias is reduced. Potentially, also additional risk factors not included in this study could be relevant. Nevertheless, given the understudied topic and lack of information on PSI + induced myelopathy/SCI are, we feel that this study provides relevant widely missing data to better understand baselines as a basic requirement for interventional studies.

## Summary

PSI + with or without myelopathy/SCI are subgroups to be evaluated on their own and represent a serious urgent, and life-threatening clinical condition. In our study, comprehensive diagnostics and a multimodal therapeutic algorithm including SCI-specific treatments were necessary. Even though when compared to the literature with mortality rates of up to 20%, the mortality rate in this study was lower (11.2%) but still substantial. Spinal infections represent a major mortality risk [3, 4]. While mortality seems not to affected by an acquired myelopathy, the economic implications and difference are striking. The costs for acute care are increased about 2.5-fold in case of an emerging PSI + -associated myelopathy/SCI.

A higher in-hospital mortality was observed in older patients and when multiple spinal segments were affected. In PSI + with SCI, improvement of neurologic symptoms occurred in 27% of the cases. In case emerging PSI + -associated myelopathy/SCI, access to early specialized SCI care is a plausible strategy to reduce complication rates, length of stay and treatment costs.

## Abbreviations

AIS: ASIA Impairment Score
ASIA: American Spinal Injury Association
BMI: Body Mass Index
CCI: Charlson Comorbidity Index
CI: Confidence Interval
CRP: C-reactive protein
EBM-level: Evidence-based medicine level
HR: Hazard Ratio
InEK: Institute for the payment system in hospitals
ICU: Intensive care unit
IQR: Interquartile range
ISA: Intraspinal abscess
ISE: Intraspinal empyema
ISNCSCI: International Standards for Neurological Classification of SCI
MRGN: Multi-resistant Gram-negative pathogens
MRSA: Methicillin-resistant *Staphylococcus aureus*
noSCI: No spinal cord injury
PSI +: Pyogenic spinal infections with intraspinal epidural involvement
SCI: Spinal cord injury
